# Mean platelet volume predicts survival in pancreatic cancer patients with synchronous liver metastases

**DOI:** 10.1038/s41598-018-24539-0

**Published:** 2018-04-16

**Authors:** Ji-bin Yin, Xin Wang, Xin Zhang, Li Liu, Rui-tao Wang

**Affiliations:** 1 Department of Gastroenterology, The Second Affiliated Hospital, Harbin Medical University, Harbin, Heilongjiang 150086 China; 2 Department of Internal Medicine, Harbin Medical University Cancer Hospital, Harbin Medical University, Harbin, Heilongjiang 150081 China; 3 Institute of Critical Care Medicine, Heilongjiang Academy of Medical Science, Harbin, Heilongjiang 150081 China

## Abstract

Most pancreatic cancer (PC) patients manifest multiple liver metastases at the time of diagnosis. Activated platelets play a key role in tumor growth and tumor metastases. Mean platelet volume (MPV) is a platelet index and is altered in patients with malignancies. This study aimed to evaluate whether MPV can effectively predict death in PC patients with synchronous liver metastases. The clinical data of 411 PC patients with synchronous liver metastases between January 1, 2006 and December 31, 2013 were retrospectively analyzed. Subjects were divided into two groups by MPV levels. Clinicopathological data were collected retrospectively and relationships between MPV levels and clinical parameters were evaluated. Survival analysis was performed. Increased MPV was not significantly correlated with tumor location, tumor size, and CA19.9. The Kaplan-Meier analysis showed that the overall survival of patients with MPV > 8.7 fL was significantly shorter than that of those with MPV ≤ 8.7 fL (log-rank p < 0.001). Multivariable Cox proportional hazards model identified MPV as an independent poor prognostic factor for overall survival. In conclusion, elevated MPV is associated with worse survival outcome in PC patients with synchronous liver metastases. Further studies are warranted.

## Introduction

Pancreatic cancer (PC) is the fourth leading cause of cancer-related death in the United States and the seventh worldwide^1,2^. Liver metastasis occurs in 70% of PC patients and the overall survival (OS) for PC patients with liver metastasis is only 11.1 months^3^. Therefore, identification of serum tumor markers of PC is urgently needed and may be helpful for convenient prognosis and treatment options.

Mounting evidence shows that platelets contribute to various steps of cancer growth, invasion, and metastasis. Thrombocytosis correlates with poor prognosis in patients with gastric, pancreatic, colorectal, ovarian, and endometrial cancer^4–8^. Since platelet count is determined by the balance between the rate of production and death of platelets, in the presence of efficient compensatory mechanisms, a normal platelet count could conceal the presence of highly hypercoagulative and pro-inflammatory cancer phenotypes^9^.

A commonly used measurement of platelet size in clinical practice is mean platelet volume (MPV). MPV is an indicator of platelet activation^10^. Alteration of MPV levels in breast, lung, gastric, colon, and ovarian cancer has been reported^11–14^. However, its clinical implication in PC patients with synchronous liver metastases has not been investigated.

The purpose of this study was to analyze the prognostic value of MPV in PC patients with synchronous liver metastases and evaluate the correlation between pre-treatment MPV levels and clinical-pathological parameters.

## Results

Among the 411 patients, the mean age was 59.6 ± 10.6 years (range 29–89). 275 (66.9%) were men and 136 (33.1%) were women.

**Figure 1:**
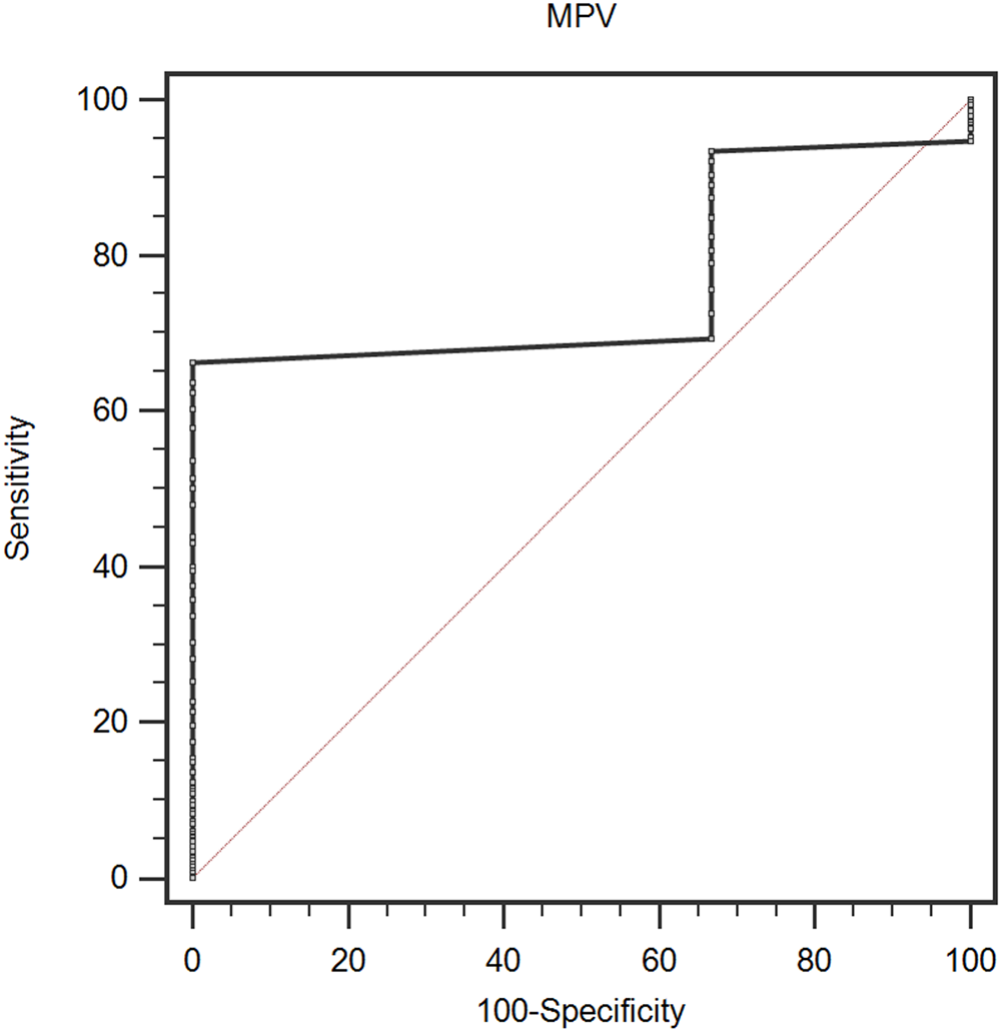
Optimized cut-off value was determined for MPV using standard ROC curve analysis.

A cutoff value of 8.7 fL (receiver operating characteristic curves constructed between death events and censors) was selected to categorize patients as high pretreatment or low pretreatment MPV. We analyzed receiver operating characteristic curve and found that elevated MPV significantly and accurately predicted PC overall survival (AUC = 0.764, 95% CI: 0.720–0.805, p = 0.004, Fig. 1**)**.

**Figure 2:**
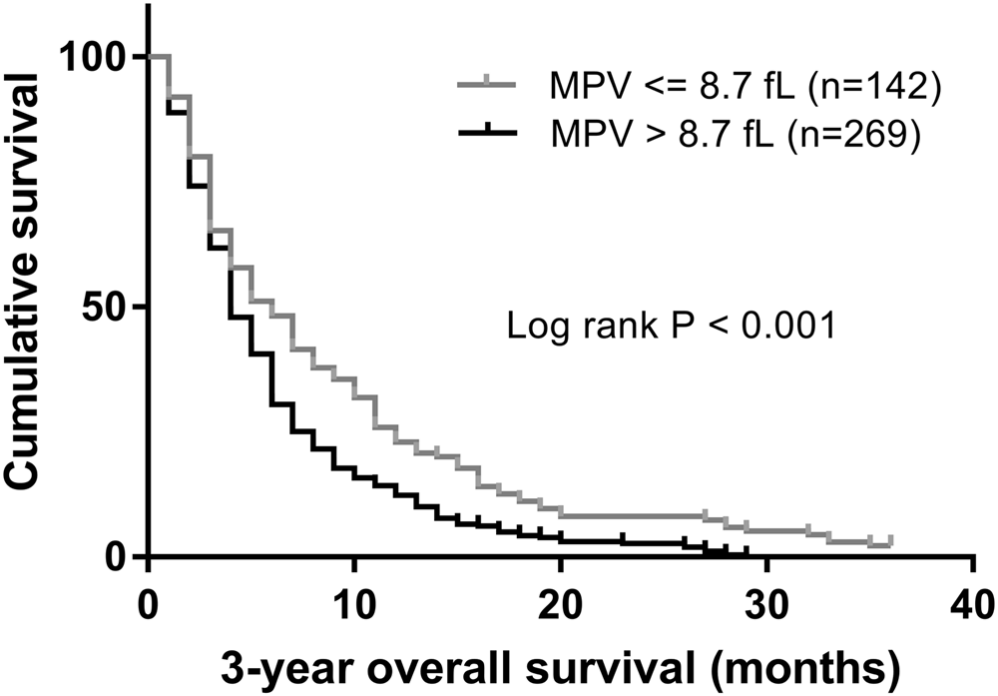
Kaplan–Meier analysis of overall survival in pancreatic cancer patients with synchronous liver metastases according to their positive and negative MPV levels around the cut-off of 8.7 fL. The median survival time (95% CI) was 4.3 (3.8, 4.7) months for 269 patients with MPV > 8.7 fL and 5.0 (3.5, 6.6) months for 142 patients with MPV ≤ 8.7 fL, respectively.

Among all patients, 269 (65.5%) had higher pretreatment MPV level (MPV > 8.7 fL). With a median follow up of 36 months, 408 (99.3%) patients had death events. 269 patients with MPV > 8.7 fL and 139 patients with MPV ≤ 8.7 fL had death events. Patients with MPV > 8.7 fL (n = 269) exhibited significantly shorter overall survival compared to those with MPV ≤ 8.7 fL (n = 142) (0.0% vs. 2.1%, p < 0.001). The median survival time (95% CI) was 4.3 (3.8, 4.7) months for patients with MPV > 8.7 fL and 5.0 (3.5, 6.6) months for patients with MPV ≤ 8.7 fL, respectively. The Kaplan-Meier OS curves of the normal versus elevated MPV showed a significant separation (Fig. 2).

The potential relationships between clinical characteristics and MPV levels are shown in Tables 1 and 2. Reduced hemoglobin and increased platelet count were more likely to appear in the lower MPV group than in the higher MPV group. However, there were no significant differences between groups based on their age, gender, BMI, smoking status, drinking status, tumor location, tumor size, and CA19.9.

**Table 1:** The relation between clinico-pathological parameters and MPV levels in PC patients with liver metastases.

Variables	Total n (%)	MPV ≤ 8.7 (fL) n (%)	MPV > 8.7 (fL) n (%)	P value
Age (years)				0.869
<65	280 (68.1)	96 (67.6)	184 (68.4)	
≥65	131 (31.9)	46 (32.4)	85 (31.6)	
Gender				0.661
Male	275 (66.9)	97 (68.3)	178 (66.2)	
Female	136 (33.1)	45 (31.7)	91 (33.8)	
Smoking history				0.669
Ever	130 (31.6)	43 (30.3)	87 (32.3)	
Never	281 (68.4)	99 (69.7)	182 (67.7)	
Drinking history				0.779
Ever	93 (22.6)	31 (21.8)	62 (23.0)	
Never	318 (77.4)	111 (78.2)	207 (77.0)	
Tumor location				0.252
Head	73 (17.8)	21 (14.8)	52 (19.3)	
Body/tail	338 (82.2)	121 (85.2)	217 (80.7)	
Tumor size				0.938
≤40 mm	195 (47.4)	67 (47.2)	128 (47.6)	
>40 mm	216 (52.6)	75 (52.8)	141 (52.4)	
CA19.9 (U/ml)				0.864
>190	306 (74.5)	105 (73.9)	201 (74.7)	
≤190	105 (25.5)	37 (26.1)	68 (25.3)	

PC, pancreatic cancer; MPV, mean platelet volume; CA19.9, carbohydrate antigen 19.9.

**Table 2:** The relation between clinical parameters and MPV levels in PC patients with liver metastases.

Variables	MPV ≤ 8.7 (fL)	MPV > 8.7 (fL)	P value
Age (years)	60.5 (10.0)	59.2 (10.9)	0.229
BMI (kg/m^2^)	21.9 (3.9)	22.2 (3.7)	0.404
Albumin (g/L)	41.2 (6.3)	40.9 (6.2)	0.653
Hemoglobin (g/dl)	125.2 (22.4)	130.7 (18.7)	0.009^*^
WBC (×10^9^/L)	8.2 (3.9)	8.5 (5.7)	0.540
Platelet count (×10^9^/L)	225.8 (86.6)	202.8 (76.3)	0.008^*^
PDW (%)	16.6 (1.2)	16.5 (2.4)	0.724

BMI, body mass index; WBC; white blood cell count; PDW; platelet distribution width. ^*^P-value indicates p-value lower than 0.05.

Cox’s regression analysis was performed on several variables, including age, sex, BMI, alcohol consumption, smoking status, tumor location, tumor size, CA19.9, chemotherapy, albumin, hemoglobin, WBC, platelet count, MPV, and PDW. The following factors were identified as posing an increased risk for PC by the univariate analysis: tumor location, CA19.9, chemotherapy, albumin, WBC, and MPV. Hemoglobin (p = 0.090) showed a weak association (Table 3). Next, all the factors with a p-value less than 0.10 in univariate analysis were included in multivariate analysis (Table 4). Multivariate analysis was performed on these factors (Table 4) and the following factors were identified as independent risk factors: tumor location, CA19.9, WBC, MPV, and chemotherapy.

**Table 3:** The univariate analysis of overall survival in PC patients with liver metastases.

	Hazard ratio	95% CI	*P*-value
Age (years) (≥65 versus <65)	1.064	0.864–1.311	0.557
BMI (kg/m^2^)	0.990	0.962–1.020	0.515
Gender (male versus female)	0.953	0.774–1.173	0.647
Smoking history (ever versus never)	0.897	0.726–1.109	0.316
Drinking history (ever versus never)	0.844	0.667–1.067	0.156
Tumor location (head versus body/tail)	1.295	1.004–1.670	0.047^*^
Tumor size (mm) (≥40 versus <40)	1.148	0.944–1.396	0.168
CA19.9 (U/ml) (>190 versus ≤190)	1.356	1.082–1.701	0.008^*^
Peripancreatic invasion (yes versus no)	0.890	0.700–1.130	0.339
Radiotherapy (yes versus no)	1.155	0.881–1.514	0.296
Chemotherapy (yes versus no)	0.718	0.585–0.882	0.002^*^
Albumin (g/L)	0.981	0.966–0.996	0.014^*^
ALT (U/L) (log-value)	1.092	0.985–1.212	0.095
AST (U/L) (log-value)	1.190	1.066–1.329	0.002^*^
ECOG performance status	1.117	0.980–1.273	0.096
Hemoglobin (g/dl)	0.996	0.991–1.001	0.090
WBC (×10^9^/L)	1.035	1.016–1.054	<0.001^*^
Platelet count (×10^9^/L)	1.000	0.999–1.001	0.736
MPV (fL) (>8.7 versus ≤8.7)	1.461	1.183–1.804	<0.001^*^
PDW (%)	1.018	0.973–1.066	0.438

ALT, alanine aminotransferase; AST, aspartate transaminase.

**Table 4:** The multivariate analysis of overall survival in PC patients with liver metastases.

	Hazard ratio	95% CI	*P*-value
Tumor location (head versus body/tail)	1.494	1.094–2.041	0.012^*^
Albumin (g/L)	0.996	0.976–1.017	0.731
CA19.9 (U/ml) (>190 versus ≤190)	1.300	1.031–1.641	0.027^*^
ALT (U/L) (log-value)	0.897	0.735–1.096	0.288
AST (U/L) (log-value)	1.187	0.944–1.492	0.142
ECOG performance status	1.059	0.929–1.208	0.392
Hemoglobin (g/dl)	0.996	0.990–1.001	0.144
WBC (×10^9^/L)	1.028	1.006–1.050	0.001^*^
MPV (fL) (>8.7 versus ≤8.7)	1.506	1.191–1.903	0.001^*^
Chemotherapy (yes versus no)	0.589	0.463–0.750	<0.001^*^

Variables that showed a p-value < 0.10 in univariate analysis were included in a multivariate Cox proportional hazards regression model.

## Discussion

This study showed that elevated MPV is associated with worse survival outcome in PC patients with synchronous liver metastases.

Despite best current medical and surgical treatment, the overall prognosis of PC patients with liver metastases remains poor. Therefore, it is of great importance to identify novel prognostic factors. There is a complex interplay between platelet-induced tumor growth and tumor cell-induced platelet activation^15^. Platelet-derived growth factor receptor beta (PDGFRβ) over-expression was observed in pancreatic cancer^16^. Moreover, PC cells are able to induce platelet aggregation via activation of thrombin^17^. The improved responses of PC xenografts to the multimodality treatment partly derived from PDGFRβ inhibition in PC patients^18^. A recent study reported platelet factor 4 as a new marker for the discrimination of PC patients and healthy controls^19^. In addition, aspirin therapy reduces the ability of platelets to promote PC cell proliferation^20^.

In agreement with the studies above, our study indirectly confirmed the findings using a simple platelet index. Moreover, our study can form the basis for further mechanistic studies and ultimately aid in patient-tailored selection of therapeutic strategies.

The mechanisms underlying the association between MPV and survival in PC patients with synchronous liver metastases have yet to be fully defined. Circulating tumor cells re-engage additional platelets, which facilitates tumor cell adhesion, arrest and extravasation, and metastasis^21^. MPV was an early marker of activated platelets. Strong evidence indicates that larger platelets are more metabolically and enzymatically active than smaller platelets. Platelets coordinate complex angiogenic responses through a number of different mechanisms, including direct cellular contact, local release of pro-angiogenic proteins into the tumor microenvironment, and recruitment of distant cells into the tumor mircroenvironment^22^. Activated platelets release secretory factors that promote chemokines, proteolytic enzymes and microparticles within the microenvironment to promote tumor cell invasion^15^. In addition, use of antiplatelet agents resulted in reductions in liver tumor nodules from human pancreatic tumor cells in nude mice^23^.

Consistent with our findings, Tuncel *et al*. found that increased MPV is a prognostic factor for metastatic colorectal cancer patients treated with bevacizumab-combined chemotherapy^24^. Gu *et al*. observed that high pre-treatment MPV level in invasive breast cancer patients associates with worse clinicopathologic features^25^. Lian *et al*. showed that a high baseline MPV level was correlated with increased metastasis^26^. These data are also consistent with the current knowledge that anti-platelet is considered to be a part of cancer adjuvant therapy^27^. Therefore, our results may help to inform treatment decisions and predict treatment outcomes.

The current study has several limitations. Firstly, as a retrospective and single-center study, the limitations of the current research lie in its intrinsic features. Additional prospective studies with larger patient numbers may provide more definitive data to clarify the significance of the findings. Secondly, the mechanisms underlying the involvement of MPV in PC patients with liver metastases remains unclear, to which further investigation should be addressed. Thirdly, the patients were composed of only Chinese. The application to other ethnic groups needs further investigation.

In conclusion, as a noninvasive, low cost, easily assessable and reproducible parameter, MPV is a promising tool for the assessment of PC prognosis in clinical practice in the future. The mechanism behind the association of elevated MPV with poorer prognosis in PC patients with synchronous liver metastases should be clarified.

## Patients and Methods

### Study population

We reviewed the clinical data of 411 consecutive PC patients with synchronous liver metastases who visited Harbin Medical University Cancer Hospital from January 1, 2006 and December 31, 2013. All patients were histologically confirmed by two experienced pathologists and did not receive any adjuvant therapy before histologically proven diagnosis. The patients with hematological disorders and medical treatment with anticoagulant, statins, and acetylic salicylic acid were excluded from our study. Overall survival (OS) was calculated from the date of diagnosis until the date of death or until the date of the last follow-up. Follow-up evaluations were performed every 3 months. The deadline for follow-up evaluations is December 31, 2016. The patients in our study will be followed-up for 36 months. The median follow-up duration is the time between the date of diagnosis and the date of death or the date of the last follow-up. Because many patients died within 12 months, their follow-up duration is not more than 12 months. The median follow-up duration in our study was 6.8 months. The pancreatic cancer tissue specimens were obtained using endoscopic ultrasound via fine-needle aspiration. All PC patients received MRI scanning of head, CT lung screening, abdominal enhanced MRI, and ECT evaluation of skeletal metastases. The patients with other organ metastasis besides live metastasis were not included in our study. Fasting venous blood samples were collected from the patients within one week prior to histologically proven diagnosis. Platelet indices were measured by an autoanalyzer (Sysmex XE-2100, Kobe, Japan) and were processed within 30 minutes after blood collection. The normal values of MPV are 7.0–11.0 fL.

This study complied with the Helsinki Declaration. The Ethics Committee of The Institutional Ethics Review Board of Harbin Medical University Cancer Hospital approved the study protocol. Because it was a retrospective study, the informed consent was waived.

### Statistical analysis

The statistical tests were carried out using SPSS version 22.0 software (SPSS Inc., Chicago, IL, USA). Normally distributed continuous variables were compared with the Student’s t test. Categorical variables were compared with the Chi-square test. OS was calculated by the Kaplan–Meier method and compared with log-rank test. The Receiver operating characteristic (ROC) curves were used to identify potential cut-off values for the CA19.9 and MPV levels, along with the sensitivities and specificities using MedCalc version 15.0. Multivariate analyses were performed using the Cox proportional hazards regression model to determine independent predictive factors of OS. Variables that reached a p-value of ≤0.10 in the univariate analysis were entered into multivariate analyses. All reported p-values were two-sided, and statistical significance was set at <0.05.
